# Towards Automated Binding Affinity Prediction Using an Iterative Linear Interaction Energy Approach

**DOI:** 10.3390/ijms15010798

**Published:** 2014-01-09

**Authors:** C. Ruben Vosmeer, René Pool, Mariël F. van Stee, Lovorka Perić-Hassler, Nico P. E. Vermeulen, Daan P. Geerke

**Affiliations:** AIMMS Division of Molecular Toxicology, Department of Chemistry and Pharmaceutical Sciences, Faculty of Sciences, VU University Amsterdam, De Boelelaan 1083, 1081 HV Amsterdam, The Netherlands; E-Mails: c.r.vosmeer@vu.nl (C.R.V.); r.pool@vu.nl (R.P.); mariel.vanstee@tno.nl (M.F.S.); lovorka.peric@gmail.com (L.P.-H.); n.p.e.vermeulen@vu.nl (N.P.E.V.)

**Keywords:** Automated binding free energy calculation, iterative LIE method, CYP 2D6, aryloxypropanolamines

## Abstract

Binding affinity prediction of potential drugs to target and off-target proteins is an essential asset in drug development. These predictions require the calculation of binding free energies. In such calculations, it is a major challenge to properly account for both the dynamic nature of the protein and the possible variety of ligand-binding orientations, while keeping computational costs tractable. Recently, an iterative Linear Interaction Energy (LIE) approach was introduced, in which results from multiple simulations of a protein-ligand complex are combined into a single binding free energy using a Boltzmann weighting-based scheme. This method was shown to reach experimental accuracy for flexible proteins while retaining the computational efficiency of the general LIE approach. Here, we show that the iterative LIE approach can be used to predict binding affinities in an automated way. A workflow was designed using preselected protein conformations, automated ligand docking and clustering, and a (semi-)automated molecular dynamics simulation setup. We show that using this workflow, binding affinities of aryloxypropanolamines to the malleable Cytochrome P450 2D6 enzyme can be predicted without *a priori* knowledge of dominant protein-ligand conformations. In addition, we provide an outlook for an approach to assess the quality of the LIE predictions, based on simulation outcomes only.

## 1. Introduction

*In silico* prediction of protein-binding affinities plays an important role in the process of drug design and development [1,2]. In the case of Cytochrome P450 enzymes (CYPs), computational modelling allows to rationalize or predict sites-of-metabolism of given substrates, but also to calculate CYP binding affinities [3–5]. Substrate binding affinity and selectivity prediction is of direct relevance to CYP metabolite prediction, because different CYPs can convert substrates differently [6]. In order to make computational methods for CYP binding affinity prediction suitable for use in an industrial setting, they should be cost efficient. Simultaneously, they should be of sufficient accuracy and yield insight into the details of molecular interaction.

Binding affinity predictions rely on the calculation of free energies of binding, accounting for enthalpic and entropic contributions to the binding of a drug to its (off-)target protein [7,8]. For proteins of large flexibility, free energy calculations should properly incorporate the dynamic nature of the protein and its active site. Molecular docking and scoring methods often fail in binding affinity predictions due to their static nature [9]. Free energy methods based on molecular dynamics (MD) simulations allow the structure of a protein-ligand complex to dynamically evolve in time [10,11]. However, in case of large conformational changes, induced-fit effects and rare events, sufficient sampling may come at great computational expenses. Especially for proteins of high malleability or plasticity, standard MD-based free energy methods are often inappropriate for (semi-)high-throughput affinity prediction. Moreover, CYPs and other proteins with a large or flexible active site are typically able to accommodate ligands in various binding orientations [12,13], which can render full sampling of possible binding orientations computationally prohibitive. The possibility of different binding modes poses an additional challenge to use of MD-based methods in a high-throughput setup, because it requires *a priori* knowledge of the probable binding orientations to start simulations from.

To include sufficient conformational sampling of protein-ligand complexes of high malleability at relatively low computational cost, an iterative Linear Interaction Energy (LIE) approach was recently introduced by Stjernschantz and Oostenbrink [13]. In LIE [14], the calculated free energy of binding of a ligand to a protein (Δ*G_calc_*) is related to the differences in nonbonded interaction energies obtained from MD simulations of the protein-ligand complex and of the unbound ligand in solution:

(1) $$\Delta G_{calc}=\alpha \;\left({\langle V_{lig-surr}^{vdW}\rangle }_{bound}-{\langle V_{lig-surr}^{vdW}\rangle }_{free}\right)+\beta \;\left({\langle V_{lig-surr}^{el}\rangle }_{bound}-{\langle V_{lig-surr}^{el}\rangle }_{free}\right)$$

In Equation (1), $\langle V_{lig-surr}^{vdW}\rangle$ is the van der Waals interaction energy between the ligand and its surrounding, either in solvent (free) or in the protein (bound). $\langle V_{lig-surr}^{el}\rangle$ refers to the corresponding electrostatic interaction energy terms, and angular brackets denote ensemble averages. Parameters *α* and *β* are optimized in a training procedure using experimental values for the binding free energy. The iterative LIE approach relies on combining results from multiple MD simulations of the protein-bound state, starting from distinct local minima on the potential energy surface of the protein-ligand complex [13,15]. Average interaction energies obtained from the different simulations are combined by assigning Boltzmann weights *W_i_* to the part of conformational space covered during protein-ligand simulation *i*. Each *W_i_* is calculated from the average interaction energies ${\langle V_{lig-surr}^{vdW}\rangle }_{i}$ and ${\langle V_{lig-surr}^{el}\rangle }_{i}$ obtained for simulation *i*, using [13,16]

(2) $$W_{i}=\frac{e^{\frac{-\Delta G_{calc,i}}{k_{B}T}}}{\sum_{i}^{N}e^{\frac{-\Delta G_{calc,i}}{k_{B}T}}}$$

In Equation (2)*k_B_* is the Boltzmann constant, *T* is the temperature of the system, and *N* is the number of independent simulations. The Δ*G_calc,i_*’s for the *N* independent simulations *i* are obtained by inserting ${\langle V_{lig-surr}^{vdW}\rangle }_{i}$ and ${\langle V_{lig-surr}^{el}\rangle }_{i}$ into Equation (1). The combined free energy of binding of the ligand is then calculated as

(3) $$\Delta G_{calc}=\alpha \sum_{i}^{N}W_{i}\;\left({\langle V_{lig-surr}^{vdW}\rangle }_{bound,i}-{\langle V_{lig-surr}^{vdW}\rangle }_{free}\right)+\beta \sum_{i}^{N}W_{i}\;\left({\langle V_{lig-surr}^{el}\rangle }_{bound,i}-{\langle V_{lig-surr}^{el}\rangle }_{free}\right)$$

In practice, various MD runs are started from distinct protein-ligand conformations, and Boltzmann weighting of the results of the separate simulations (Equation (2)) ranks them according to the contribution of the part of conformational space covered during simulation. Running multiple MD simulations and using Equations (2) and (3) was recently shown to yield binding affinities within experimental accuracy for highly malleable proteins such as CYPs 2C9 and 2D6 [13,15], whereas lower accuracy was reached using Equation (1) and running a single MD simulation per ligand (in which possible conformational rearrangements could not sufficiently be sampled). Thus, distinct parts of conformational space were found to contribute to the binding affinity of these flexible proteins, and free energy methods based on MD simulations starting from a single protein-ligand conformation will have difficulties in predicting binding free energies in such cases, even when using advanced methods (see e.g., reference [17]) that aim on carefully selecting and refining the starting configuration.

The above described LIE scheme shows great potential for high-throughput use, because simulations starting from different conformations can be run independently, which makes the method suitable for (massive) parallellization. Moreover, when relevant protein conformations and ligand orientations are available to start simulations from, sampling is partly accounted for *a priori*, thereby reducing computational efforts (compared to running a single MD simulation per protein-ligand complex where visiting all relevant regions of phase space is highly improbable). In the current study, we present a method to broaden the applicability of the iterative LIE approach towards automated binding affinity prediction.

A major challenge in using the iterative approach in a (semi-)high-throughput manner is proper selection of a limited number of starting structures that represent distinct and relevant parts of conformational space. Thereby, preselection of dominant protein conformations must properly include induced-fit effects, whereas selection of possible binding orientations for the ligand should ensure that relevant poses are included as well. In previous work [15], we showed that for the very flexible CYP 2D6 enzyme, relevant protein structures could be chosen based on a plasticity model of Hritz *et al.* for site-of-metabolism prediction [18]. Starting conformations of the ligand in the active site for use in LIE simulations were until now chosen based on experimental information on the site-of-metabolism [19] or based on visual inspection of obtained docking poses [13,15,20]. In the current work, we present a generic method to train and use the LIE models for binding affinity prediction. In this automated procedure, possible ligand-binding orientations are selected based on clustering of binding poses obtained from molecular docking. As a result, no user input or external knowledge is required for pose generation, and MD starting orientations for the ligand are obtained that cover different parts of conformational space (in terms of binding positions and orientations). Using the generated starting configurations, MD simulations are subsequently run in an automated way. Automation of the ligand-pose selection and MD setup is required to make the method available for generic use. Moreover, it greatly facilitates extensive training and testing of our LIE models (in the case of large availability of experimental data).

Here, we show the potential of our automated implementation by developing an iterative LIE model for CYP 2D6 binding of a series of aryloxypropanolamine analogs, for which no structural information on the protein-ligand complexes is available. In addition, we provide an outlook on how to assess the quality of the results obtained by our models. In practice, a LIE model is based on training data, comprising experimental and simulation data. Binding affinity prediction is then based on the assumption that the model is valid for a substantially larger set of ligands. A quantification of the accuracy of a binding affinity prediction would be of great value to assess if this assumption can be made. We address this issue by inspecting model behavior (in terms of predictive accuracy) under permutations of ligands between the subsets of training and test compounds used, and its relation to data obtained from simulation.

## 2. Computational Methods

In the following we describe the automated procedure for training iterative LIE models (Section 2.1) and for performing LIE predictions (Section 2.2), as well as the technical details of the protocol, which involves generation of starting structures for MD, performing MD, and combining computed interaction energies into binding free energies (Section 2.3).

### 2.1. Automated Training

**Figure 1.**
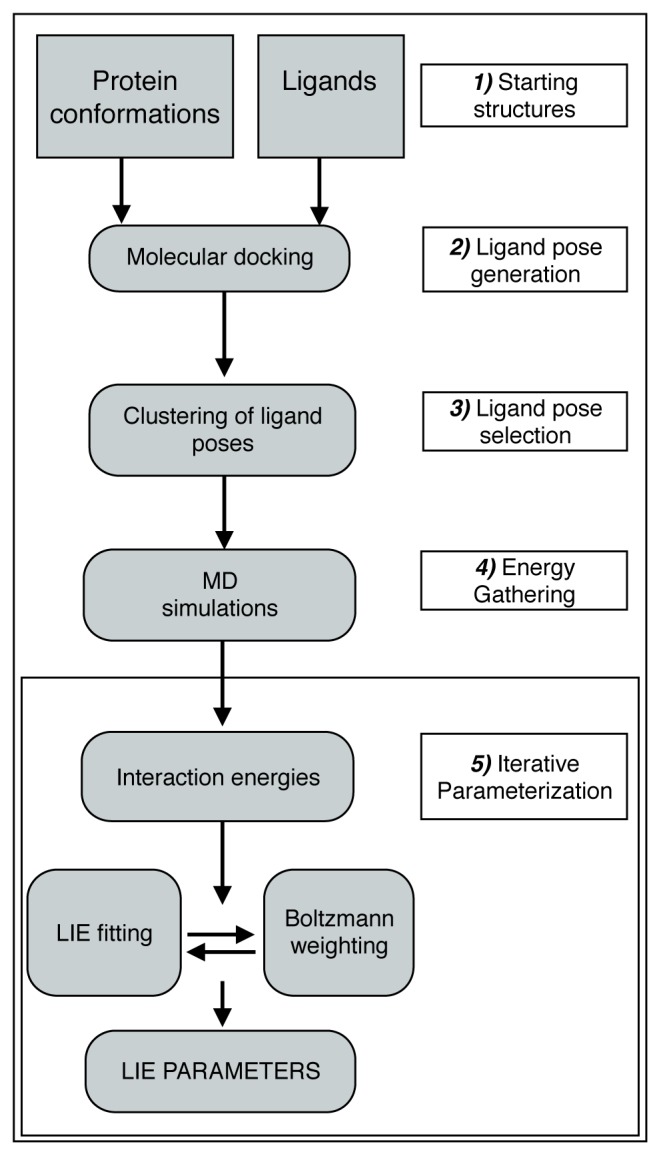
Workflow for training of iterative LIE models. In the current work, 2 protein conformations and 9 training and 8 test ligands were used to generate maximally 600 docked poses per ligand, from which 3 poses per ligand and per protein conformation were selected to start MD.

The upper part of Figure 1 (steps **1**–**4**) depicts our automated procedure for generating starting conformations and for running the MD simulations. Training (parameterization) of an iterative LIE model covers five steps (steps **1**–**5** in Figure 1). After defining the protein of interest and selecting series of training compounds for which binding free energies are experimentally known (ideally covering a broad range of affinities), one or more protein conformations are selected (Figure 1, step **1**). These protein structures will be used as templates for docking and to start independent MD simulations from, and they should be selected such that relevant parts of conformational space are covered, based on available experimental (crystal) structures or computational studies to identify representative conformations [18]. For every selected protein structure, a set of possible ligand-binding poses is generated by means of molecular docking (Figure 1, step **2**). From this set, representative binding orientations are selected based on conformational clustering (Figure 1, step **3**) using a nearest-neighbor clustering method [21,22]. Subsequently, every combination of protein structure and obtained pose therein is used as starting conformation to start an independent MD simulation from, in order to compute ensemble-averaged electrostatic and van der Waals interaction energies (Figure 1, step **4**). To run the MD simulations, an automated procedure was implemented to successively energy minimize and solvate the protein-ligand complex, add counterions to neutralize the net protein charge, thermally equilibrate the system, start production MD simulations, and retrieve average interaction energies. The same MD protocol was used to perform simulations of the unbound ligand in water. Note that ligand topologies for use in MD were generated manually in the current work (consistent with the chosen protein force field), but ongoing efforts by others [23–26] to develop automated topology builders for (drug-like) compounds already allow for full automatization of our LIE workflow. In a final step (Figure 1, step **5**), average nonbonded interaction energies obtained from the different simulations, and experimental estimates for the binding free energies are used to calibrate the LIE model parameters *α* and *β* in Equation (3), and to iteratively obtain the contributions *W_i_* (Equation (2)) of the different MD simulations [13].

### 2.2. Automated Free Energy Prediction

The calculation of Δ*G_calc_* using a predefined LIE model uses a similar setup as described in Section 2.1. Ligands are docked in the preselected protein conformations and docking poses are clustered, after which interaction energies are gathered from independent MD simulations that start from the obtained structures of the ligand-protein complex (steps **1**–**4** in Figure 1). Step **5** in Figure 1 is replaced by direct calculation of Δ*G_calc_* from weighted average interaction energies for the different simulations, using Equations (2) and (3), and the (precalibrated) LIE parameters *α* and *β*.

### 2.3. Computational Details

Using our automated procedure, an iterative LIE model was developed for the prediction of binding affinities to CYP 2D6 for a series of aryloxypropanolamine analogs. The sets of training and test compounds are presented in Tables 1 and 2, respectively. The quality of any LIE model relies heavily on the consistency of the experimental data used for training. Here, experimental binding free energies were extracted from a single inhibition study [27], and derived from reported *IC*_50_ values using the Cheng-Prusoff equation [28]:

(4) $$\Delta G_{exp}≅RT\text{ln}\;(K_{i})=RT\text{ln}\left(\frac{IC_{50}}{1+[S]/K_{m}}\right)$$

with *K_i_* the inhibition constant for inhibition of conversion of a 3-[2-(*N*,*N*-diethyl-*N*-methylamino) ethyl]-7-methoxy-4-methylcoumarin (AMMC) substrate with concentration [*S*] = 1.5 μM [27], and *K_m_* = 0.5 μM [29]. Training and test compounds were chosen such that a wide range of *IC*_50_ values was covered, ranging from 0.03 to 100 μM (corresponding to Δ*G_exp_* values between −48.2 and −27.3 kJ mol^−1^).

#### 2.3.1. Selection of Protein Conformations

As in our previous CYP 2D6 LIE study [15], selection of protein conformations (to start docking and simulation from) was based on a combined MD/virtual screening study by Hritz *et al.* [18]. Hritz identified two distinct conformations that can be used as templates for accurate docking-based site-of-metabolism prediction. Using a simple binary-decision tree based on the molecular weight of a substrate, it is decided which of the protein structures to dock the ligand into [18]. A major difference between the two CYP 2D6 structures in the decision tree is the orientation of phenylalanine 483 within the active site, which is part of a flexible loop. This active-site loop adopts substantially different orientations in the two isoform conformations, as depicted in Figure S1 of the Supplementary Material. In one of the conformations (referred to as PPD 70 by Hritz *et al.* [18], and as PPD70 in the current work), the Phe483 side chain is occupying part of the active site (by adopting a C*_α_*–C*_β_* dihedral angle *χ*_483_ of approximately 70°). In contrast, in the other conformation (referred to as CHZ 170 by Hritz, and as CHZ170 in the current work), Phe483 is opening up the active site (with *χ*_483_ *~* 170°). In order to improve the accuracy of site-of-metabolism prediction of CYP 2D6 substrates, Hritz found that the PPD70 structure is suitable for use as a template to dock substrates of low molecular weight in [18], whereas the CHZ170 conformation should be used for docking of substrates of high molecular weight. These findings indicate that reorientation of the Phe483 loop and Phe483 side-chain rotation allow CYP 2D6 to accommodate ligands of different size. Here, we use both PPD70 and CHZ170 as starting conformations of independent MD simulations, because sampling of this conformational change in a single MD simulation is computationally expensive.

#### 2.3.2. Docking Procedure

To improve sampling of available ligand-conformational space within the binding site during docking, six different sets of atomic coordinates were used for every ligand to start docking runs from. The coordinate sets were generated by rotating an arbitrary ligand conformation by *±*90° around the three Cartesian axes. The six sets of ligand coordinates were docked into the PPD70 and CHZ170 templates using the GOLD (Genetic Optimization for Ligand Docking) software (version 4.0) [30], together with the ChemScore scoring function [31]. The maximum allowed operations for the genetic docking algorithm was 50,000, with a population of 100 genes. Only the 50 best scoring poses were retained for every docking run, yielding in total maximally 300 docking poses for every combination of protein conformation and ligand. During docking, the active site of the protein was defined as a sphere with a 2.5 nm radius. The center of this sphere was positioned within the active site, at a distance of 0.2 nm from Fe along the vector connecting the heme iron and the sulphur atom of the Fe-coordinated cysteine (Cys443). For docking of the test compounds, the distance between Fe and the center of the docking sphere was increased to 0.6 nm, and the radius of the sphere was reduced to 1.0 nm.

#### 2.3.3. Clustering

For every protein conformation and ligand, binding poses to start MD from were selected from the docked poses by means of nearest-neighbor clustering [21], using clustering analysis tools included in the GROMOS11 software package [32]. The allowed number of clusters was set to five, allowing the inclusion of various starting configurations for MD in the LIE model without heavily increasing the computational cost by the number of MD simulations to be carried out. The central structures of the three most populated clusters were combined with the protein coordinates, to serve as starting structure for use in the automated MD procedure. Central structures were defined as having the smallest average root-mean-square structural deviation from other members in the cluster.

#### 2.3.4. MD Simulations

Energy minimization, preparation of the simulations, MD runs and post-MD analysis were carried out using the GROMOS11 software package [32]. During energy minimization and MD, the protein, ligands and counterions were described using the GROMOS 45A4 force-field parameter set [33]. First, the selected protein-ligand complex structures were energy minimized in vacuum using the steepest-descent method, after which they were solvated in rectangular boxes of circa 18,400 water molecules, with box lengths of approximately 8.6 nm, using a minimum solute-wall distance of 0.8 nm. The minimum distance between solute and solvent atoms was set to 0.24 nm, and the minimum distance between solvent atoms and active site or ligand atoms was set to 0.35 nm. Six sodium counterions were added to neutralize the net charge of the protein. The system was energy minimized while keeping positions of protein and ligand atoms restrained using a harmonic force constant of 2.5 *×* 10^4^ kJ mol^−1^ nm^−2^.

Subsequently, atomic velocities were randomly assigned from a Maxwell-Boltzmann distribution, and thermal equilibration was carried out in six MD simulations of 20 ps each, at temperatures of 50, 100, 150, 200, 250, and 300 K, respectively. During equilibration, positions of the protein atoms were kept restrained, using a harmonic force constant of 25,000, 2500, 250, 25, 2.5, and 0 kJ mol^−1^ nm^−2^, respectively. The equilibration was followed by a (unrestrained) 2 ns production run at 300 K, during which average nonbonded interaction energies between the ligand and its surrounding were gathered. To prevent conformational overlap between simulations starting from different protein conformations, the dihedral angle *χ*_483_ of Phe483 was kept restrained to its starting value using a harmonic potential with a force constant of 30 kJ mol^−1^ deg^−2^ [15].

Water was descibed by the SPC model [34]. The temperature in all simulations was kept constant using a Berendsen thermostat [35] with a coupling time of 0.1 ps. In the production simulations the pressure was kept constant at 1 atm using a Berendsen barostat [35] with a coupling time of 0.5 ps. The isothermal compressibility was set to 4.575 *×* 10^−4^ (kJ mol^−1^ nm^−3^)^−1^. In all simulations a time step of 2 fs was used, and bonds were constrained using the SHAKE algorithm with a geometric tolerance of 10^−4^ [36]. Nonbonded interactions were computed every time step for pairs of atoms that are stored in a charge-group based pair list and that are within a radius of 0.8 nm. Along with the pair-list update, intermediate-range interactions (between 0.8 and 1.4 nm) were computed every fifth time step and kept constant in between. Long-range electrostatic interactions (beyond 1.4 nm) were represented by a reaction field [37], using a dielectric constant of 61 [38]. Atomic coordinates were stored for analysis every 2500 time steps, and interaction energies were stored every 50 time steps.

Following our findings in a recent CYP LIE study [15], all simulations of the protein-ligand complexes were run twice (starting from identical atomic coordinates but different randomly assigned velocities) in order to improve convergence of the interaction energies ${\langle V_{lig-surr}^{vdW}\rangle }_{bound,i}$ and ${\langle V_{lig-surr}^{el}\rangle }_{bound,i}$ in Equation (3). The corresponding pairs of simulations were combined afterwards, preceeding the calculation of the average ligand-surrounding interaction energies. In summary, twelve protein-bound simulations were performed per ligand (starting from two different protein template structures and three different ligand binding orientations, with two different sets of random atomic velocities assigned).

To evaluate the average ligand interaction energies of the unbound ligands in water $\left({\langle V_{lig-surr}^{vdW}\rangle }_{free}\;\text{and }{\langle V_{lig-surr}^{el}\rangle }_{free}\right)$, every ligand was simulated in water as well. For this purpose, the solute was solvated in a cubic box filled with approximately 4500 SPC water molecules, with a box length of approximately 5 nm [34]. No counterions were added. After energy minimization, 5 ns production simulations of the ligand in water were started at 300 K. MD settings were identical to the ones described for the production MD simulations of the protein-ligand complex.

## 3. Results and Discussion

### 3.1. Automated Binding Free Energy Prediction

**Figure 2.**
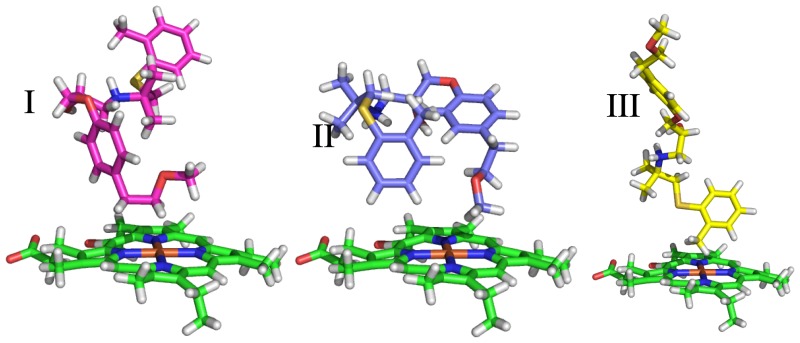
Diversity in ligand binding orientations with respect to the CYP 2D6 heme moiety (in green), as typically obtained from the automated docking and clustering procedure (steps **1**–**3**, Figure 1). Here, central structures are shown for the three most populated clusters of docking poses obtained for ligand 8 in protein template CHZ170.

The compounds of the training set were docked and clustered as described in Sections 2.3.2 and 2.3.3. The automatically selected poses typically show diversity in terms of molecular conformation and in the orientation of the principal axes of the molecules within the active site, as illustrated in Figure 2 and by the root-mean-square atom-positional deviations between pairs of obtained binding poses, which are presented in Table S1 of the Supplementary Material. For some ligands, clusters of poses outside the active site were obtained. This concerned the third most populated cluster of ligand 7 in PPD70 and the second and third most populated clusters of ligands 8–10 in PPD70. These clusters were discarded in further development of the LIE models. As we will discuss later, the reduced number of independent simulations for these ligands does not significantly affect the accuracy of the binding free energy calculations.

**Figure 3.**
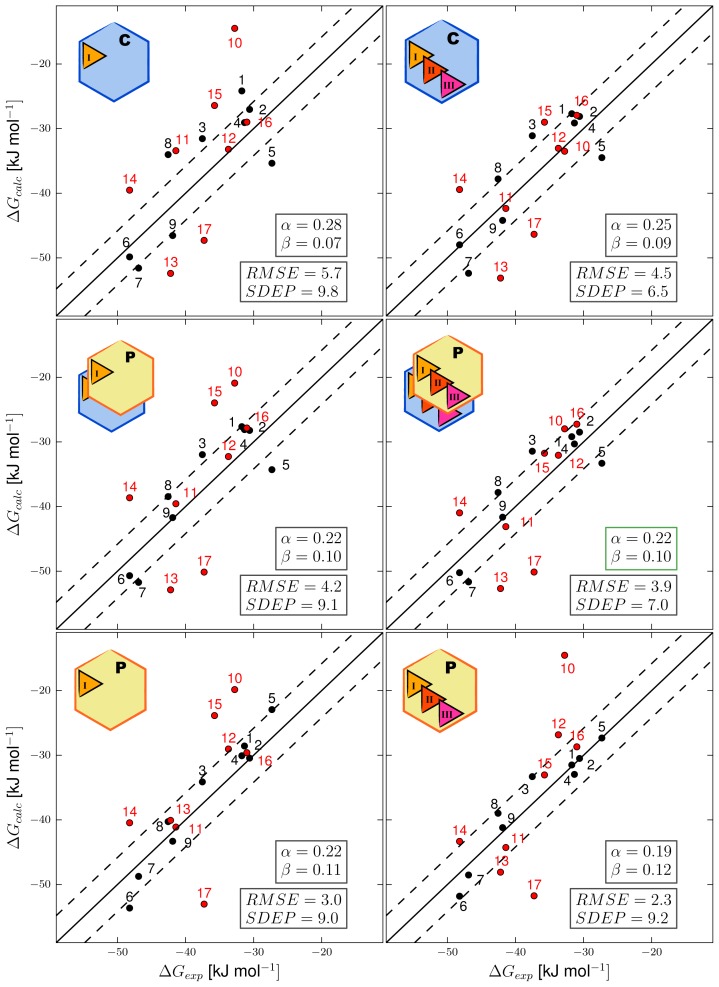
Correlations obtained using different CYP 2D6 LIE models for prediction of aryloxypropanolamine binding affinities, between the experimental (Δ*G_exp_*) and predicted free energy of binding (Δ*G_calc_*). Ideal correlation is indicated by the solid line, the dashed lines represent deviations from experiment of 1 kcal mol^−1^ (4.184 kJ mol^−1^). Training compounds are indicated in black, compounds of the test set in red. The three left models are based on simulations starting from a single ligand starting pose, while the three right models include results from simulations starting from three different starting poses. The top-row models are based on results of simulations starting from the protein conformation CHZ170 (C). Models in the bottom row include simulations with the PPD70 protein template (P). Models in the middle row include results of both P and C simulations. The LIE parameters (*α* and *β*), as well as root-mean-square errors (RMSE) and standard deviation of prediction errors (SDEP) of each model (in kJ mol^−1^) are given in de bottom-right corner of each plot.

**Figure 4.**
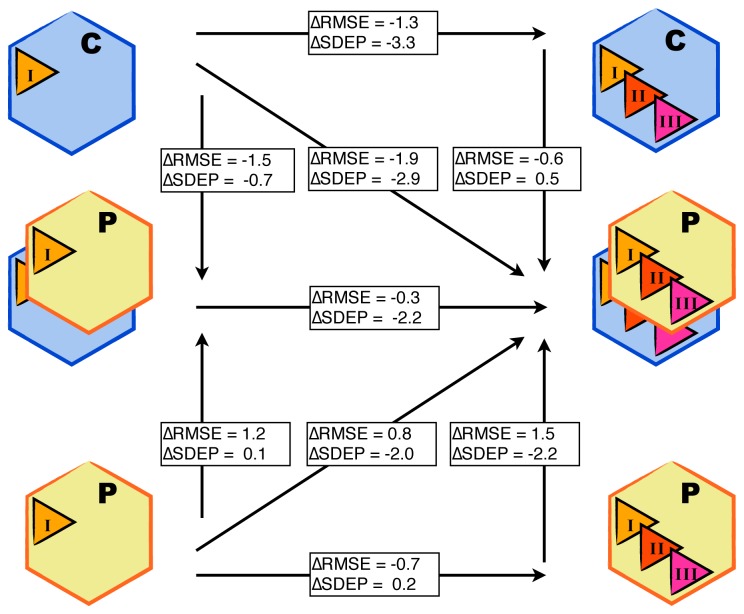
Differences (Δ, in kJ mol^−1^) in root-mean-square errors (RMSEs) and standard deviation of prediction errors (SDEPs) between the LIE models presented in Figure 3. The models on the left include results of MD simulations starting from a single active site binding orientation of the aryloxypropanolamine compounds (from the most populated clusters I), models on the right include results of MD simulations started from three different starting poses (originating from clusters I, II and III). The top-row models are based on results of simulations starting from protein conformation CHZ170 (C). Models in the bottom row include simulations starting from the PPD70 protein template (P). Models in the middle row include results of P and C simulations.

By combining results from MD simulations starting from both protein conformations (CHZ170 and PPD70) and all docking poses, a LIE model was obtained using our automated setup with *α* = 0.22 and *β* = 0.10. The correlation between calculated and experimental binding free energies using this model is displayed in Figure 3, middle-right panel. The root-mean-square errors with respect to experiment (RMSEs) of the predicted binding free energies for the training compounds (1–9 in Table 1 and Figure 3) is 3.9 kJ mol^−1^, which is within experimental accuracy. The standard deviation of prediction errors (SDEPs) of the test set of compounds (10–17 in Table 2 and Figure 3) for the combined LIE model is 7.0 kJ mol^−1^, which corresponds to an inaccuracy of one order of magnitude in terms of predicted inhibition constants (Equation (4)). Individual errors in the predicted binding free energies of the test and training compounds are reported in Table S1 of the Supplementary Material. For comparison, Figure 3 also presents LIE models obtained using results of MD simulations starting from a single protein conformation (top and bottom panels, for CHZ170 and PPD70, respectively) or from a single ligand starting pose (i.e., the central structure of the most populated cluster of docking poses, left panels). In accordance with the findings of Stjernschantz and Oostenbrink [13] and of us [15] in recent LIE studies on thiourea binding to CYP 2C9 and 2D6, the accuracy of our LIE model improves when including results from MD simulations starting from different ligand binding poses (*cf.* left panels and right panels in Figure 3): the RMSEs and SDEPs decrease when compared with LIE models that do not include all simulations, see horizontal arrows in Figure 4. As mentioned above, less than three starting poses were used for ligands 7–10 in all models, but this does not influence the predictive quality for these compounds. For example, for the LIE models based on the PPD70 template (bottom panels in Figure 3) and for those using the CHZ170 and PPD70 templates (middle panels in Figure 3), experimental accuracies were obtained for ligands 7–9 upon including two additional PPD70 binding poses for the other ligands into the models. In addition, when starting from the CHZ170-based LIE model (Figure 3, upper right panel) and including three simulations starting from the PPD70 template for all ligands except for ligands 7–10 (for which fewer PPD starting structures are available), calculated binding free energies for ligands 7–10 are still within experimental accuracy, see middle-right panel of Figure 3.

**Figure 5.**
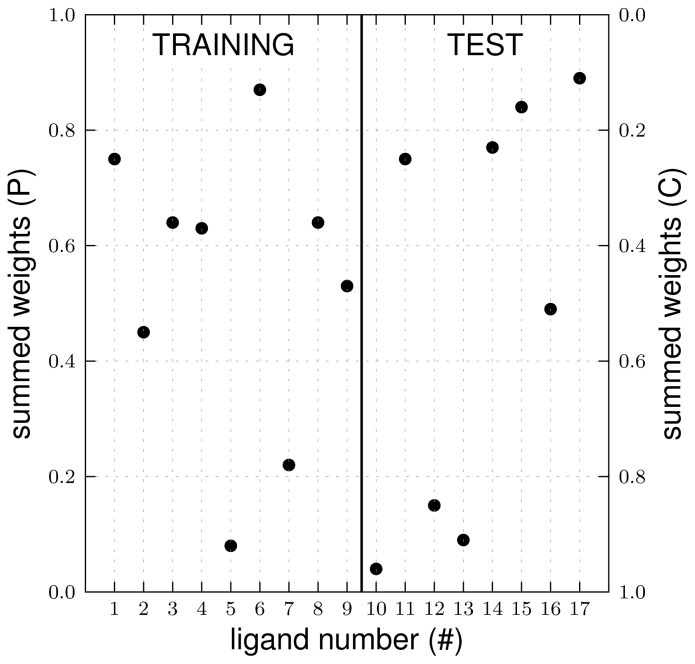
Summed Boltzmann weights of the simulations starting from either PPD70 (P) or CHZ170 (C) template structures for the aryloxypropanolamines (numbered 1–9 for the training set, 10–17 for the test set) within the final CYP 2D6 LIE model, which includes results of simulations starting from both the PPD70 and CHZ170 template structures.

Because of the significantly lower SDEP (and RMSE) values of the LIE models including simulations starting from several ligand poses (when compared to models based on single ligand poses, Figures 3 and 4), only these models are considered in the remaining. Figures 3 and 4 demonstrate that the predictive quality of the LIE models also depends on the choice of the protein template to start docking and MD from. For the considered class of compounds, the SDEP is significantly lower for the CHZ170 based model (upper right panel of Figure 3) than for the PPD70 based model (Figure 3, bottom-right panel). The combined LIE model (using both protein templates) is also more predictive than the model based on PPD70 simulations only (as shown by a lower SDEP, Figure 4). In terms of observed SDEPs and RMSEs, the combined LIE model and the CHZ170-based model show comparable accuracy. As demonstrated in Figure 5, both the PPD70 and the CHZ170 simulations contribute to the binding free energies predicted by the combined model. For many ligands, the sum of the Boltzmann weights *W_i_* of the PPD70 simulations adds up to significant values, Figure 5. This shows the relevance of including results from simulations started from both the CHZ170 and the PPD70 templates into the fully combined LIE model. These findings are in accordance with our recent LIE study on thiourea binding to CYP 2D6 [15], in which closest agreement with experiment was obtained when combining results from MD simulations starting from two protein structures (and from two distinct ligand-binding poses that were selected based on visual inspection).

From the above, when comparing our CYP 2D6 LIE models based on MD simulations starting from either single or both protein template structures, we found that the predictive ability of the LIE model for the aryloxypropanolamines is mostly dependent on inclusion of simulations starting from the CHZ170 template. On the other hand, in our recent LIE study on CYP 2D6 binding to the smaller thioureas [15], the model accuracy for the thioureas was found to be mostly depending on inclusion of simulations starting from the PPD70 template. This difference is in line with the results of Hritz *et al.*, who found that the accuracy of docking-based site-of-metabolism prediction for CYP 2D6 substrates improves when using the PPD70 template for substrates of small molecular weight (*<*280 g mol^−1^), and the CHZ170 template for docking of large substrates [18].

### 3.2. Assessing the Predictive Quality of the LIE Models

**Figure 6.**
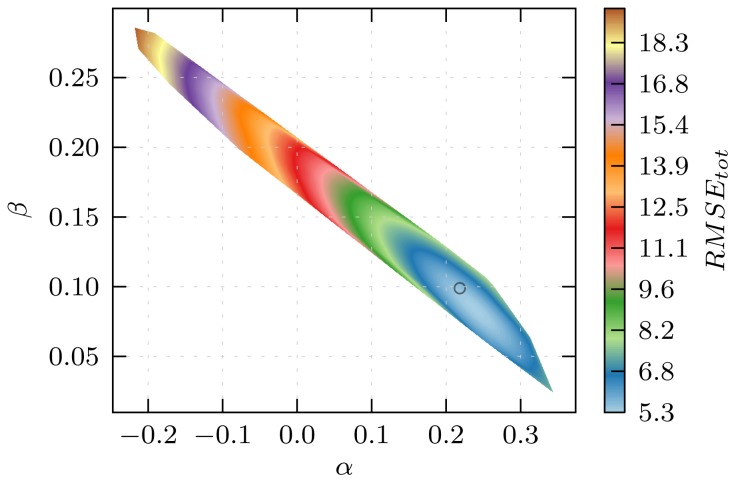
*RMSE_tot_* (root-mean-square error with respect to experiment) in kJ mol^−1^ for all 17 predicted binding free energies, as a function of model *α* and *β* parameters. The data in this figure have been generated by permuting over all possible combinations of training sets of size 9 out of the total number of ligands (17), and by optimizing the LIE *α* and *β* parameters for each permutation. The center of the circle refers to the model in the middle-right panel of Figure 3, for which *RMSE_tot_* = 5.5 kJ mol^−1^.

In developing the LIE models discussed in Section 3.1, the choice of ligands that constitute the training set was based on chemical knowledge, aiming on obtaining maximal spread in terms of experimental binding free energies and structural diversity within the sets of compounds used to train (and test) the model. We tested the suitability of our choice for dividing the set of 17 aryloxypropanolamines into a training and a test set, by calibrating and evaluating LIE models for all combinations of test and training sets (*i.e.*, for all 24,310 permutations between the sets of 9 training and 8 test compounds). From Section 3.1, the permuted LIE models include results from all simulations (*i.e.*, from any of the starting protein-ligand conformations). As a measure of the predictive quality of the permuted LIE models, we use the total root-mean-square errors *RMSE_tot_* for all 17 (Δ*G_exp_,*Δ*G_calc_*) data points in the generated models. In Figure 6, *RMSE_tot_* of all permuted models (as a function of the corresponding *α* and *β* parameters) is shown. For the optimal combination of test and training set, *RMSE_tot_* is minimal. For this combination, we found a smaller spread in experimental binding free energies and molecular weights within the training set (comprising ligands 3, 4, 6, 7, 8, 10, 15, 16, 17) when compared to the originally chosen training set (Table 1). This indicates that for training of the CYP 2D6 binding affinity model for the aryloxypropanolamines considered, use of chemical knowledge did not lead to the optimal choice in terms of training and test compounds. However, Figure 6 also shows that the combined model described in Section 3.1 has *α* and *β* close to their optimal values, indicating that using chemical knowledge did indeed yield a model that allows for sufficiently accurate predictions of the binding free energies of the ligands (*RMSE_tot_* = 5.5 kJ mol^−1^) when compared to the optimal LIE model in terms of the division between training and test set (*RMSE_tot_* = 5.3 kJ mol^−1^).

Our comparison of permuted LIE models gives direction in assessing the applicability of LIE models to (accurately) predict binding free energies for compounds for which no experimental estimates of the binding affinity are available. Ultimately, the quality of LIE predictions can only be validated by comparison to experiment. Here, we examine if prediction accuracy (model applicability) can also be assessed by comparing simulation results for compounds with unknown affinity, to the spread in simulation data obtained for the training set. When comparing *RMSE_tot_* values for all permutations of LIE models that include outcomes of simulations starting from the PPD bound structures only, inclusion of compounds 10 and 17 into the training set was found to result in large differences in *RMSE_tot (_*by more than 2.5 kJ mol^−1^) when compared to the permuted model with lowest *RMSE_tot_* (5.1 kJ mol^−1^, data not shown). This is probably due to the low predictivity of the set of PPD70 simulations for the binding free energy of compounds 10 and 17 (Figure 3, bottom-right panel). A comparison of the (weighted) outcomes of the PPD70 simulations for the 17 aryloxypropanolamines showed that values for the right-hand terms in Equation (3) as calculated for test or training compounds with strongly deviating binding free energy predictions (such as compound 10) are situated away from the model’s centroid in the corresponding [ $\alpha (V_{bound}^{vdW}-V_{free}^{vdW}),\beta (V_{bound}^{el}-V_{free}^{el})$] coordinate system, by more than twice the variance along at least one of the model principal axes (PAs) as determined in a Mahalanobis or principal component like analysis (Figure S2). Although trivial, it is important to note that this analysis is not meaningful in the [Δ*G_exp_,*Δ*G_calc_*] coordinate system, because no Δ*G_exp_* values are available in practice for the predictions of interest. These observations suggest that molecular simulation data can be used to assess prediction accuracies for new compounds, where a model’s applicability region is defined by the variances along the PAs (i.e., by the spreads in calculated interaction energies as scaled by *α* and *β* that was obtained for the training compounds).

After inspection of the variances along the PAs for all permuted models based on the simulations starting from the PPD70 and CHZ170 template structures, we were unable to detect strongly deviating free energy predictions with high precision. This is due to (i) the limited number of compounds used here to train and test the models; and (ii) the fact that the LIE models including simulations starting from both the PPD70 and CHZ70 template structures allow for more accurate predictions (which again underscores the importance of including protein plasticity in constructing LIE models). To further explore the possibility of defining model applicability by the above approach based on simulation data only, larger data sets will be examined in near future.

## 4. Conclusions

In the current work, we have shown that the iterative LIE approach as recently introduced for improved binding affinity prediction to flexible proteins such as Cytochrome P450s [13,15] can be used to predict binding free energies in a generic way. For this purpose, an automated workflow was designed using preselected protein conformations, ligand-docking and clustering, and a generic set-up to run MD simulations. For a set of aryloxypropanolamines we demonstrated that using this workflow, CYP 2D6 binding affinities can be predicted without *a priori* knowledge of dominant conformations of the protein-ligand complex. Our automated LIE calculations reconfirmed the importance of including different MD simulations starting from distinct relevant protein-ligand conformations in binding affinity prediction for flexible proteins. In addition, we discussed a method to assess the predictive quality of our LIE models based on simulation outcomes only.

## Supplementary Information



## Figures and Tables

**Table 1.** Compounds used in training of the CYP 2D6 LIE model for aryloxypropanolamines. Experimental values for *IC*_50_’s (reported by Vaz *et al.* [27]) and derived binding free energies (Δ*G_exp_*) are given, as well as Molar masses (M) and net charges of the compounds (q).

Ligand number # (# in Vaz *et al.*)	Structure	Properties
1 (6)	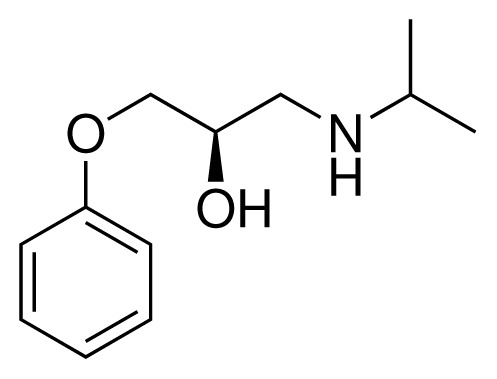	*IC*_50_ = 18 μM Δ*G_exp_* = −31.73 kJ mol^−1^ M = 210.30 g mol^−1^ q = +1*e*
2 (10)	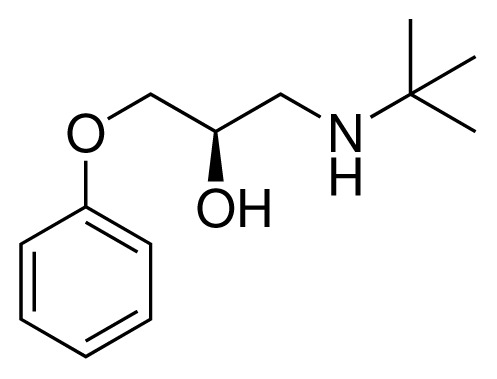	*IC*_50_ = 28 μM Δ*G_exp_* = −30.59 kJ mol^−1^ M = 224.32 g mol^−1^ q = +1*e*
3 (7)	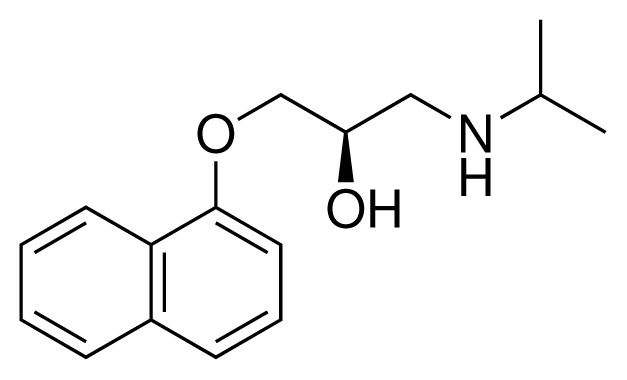	*IC*_50_ = 1.9 μM Δ*G_exp_* = −37.53 kJ mol^−1^ M = 260.36 g mol^−1^ q = +1*e*
4 (3)	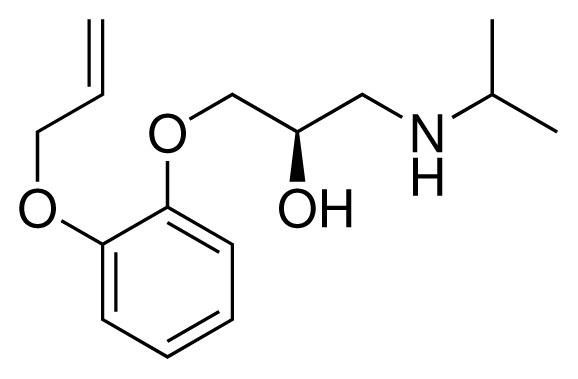	*IC*_50_ = 21 μM Δ*G_exp_* = −31.34 kJ mol^−1^ M = 266.36 g mol^−1^ q = +1*e*
5 (8)	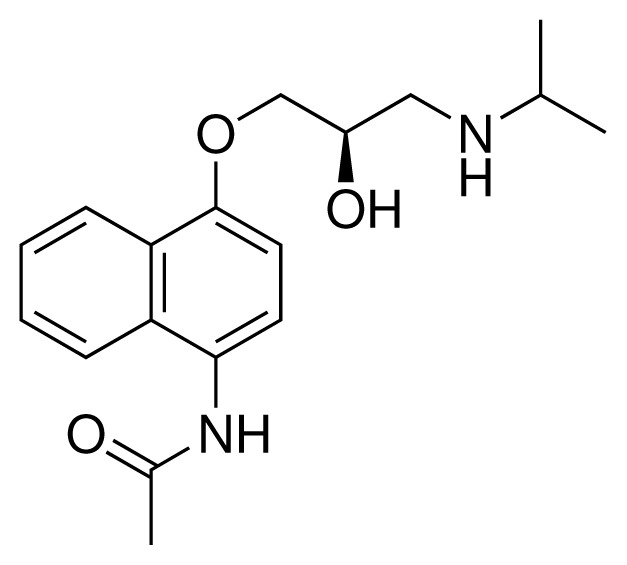	*IC*_50_ = 100 μM Δ*G_exp_* = −27.31 kJ mol^−1^ M = 317.41 g mol^−1^ q = +1*e*
6 (5)	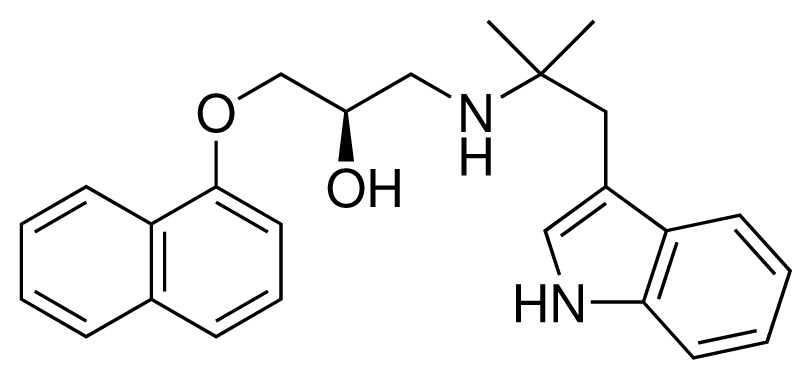	*IC*_50_ = 0.03 μM Δ*G_exp_* = −48.22 kJ mol^−1^ M = 389.52 g mol^−1^ q = +1*e*
7 (26)	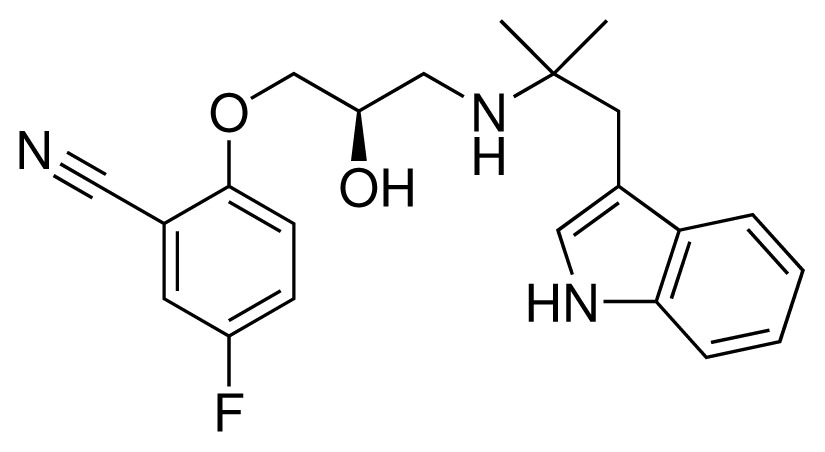	*IC*_50_ = 0.05 μM Δ*G_exp_* = −46.90 kJ mol^−1^ M = 382.46 g mol^−1^ q = +1*e*
8 (22)	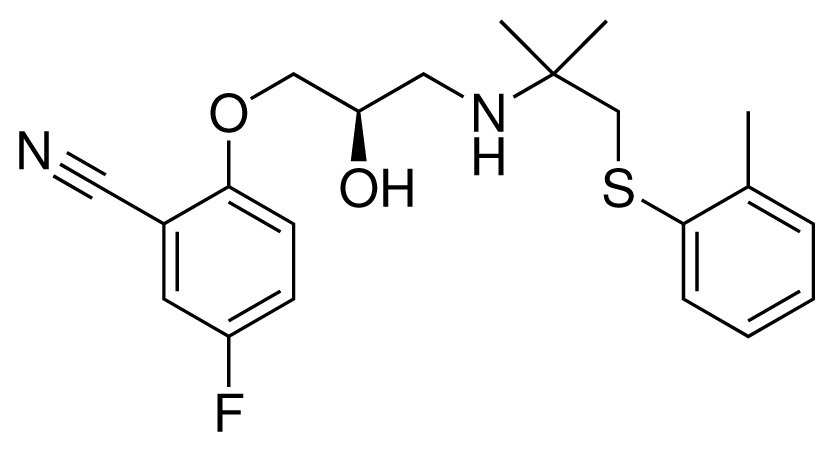	*IC*_50_ = 0.28 μM Δ*G_exp_* = −42.56 kJ mol^−1^ M = 404.60 g mol^−1^ q = +1*e*
9 (28)	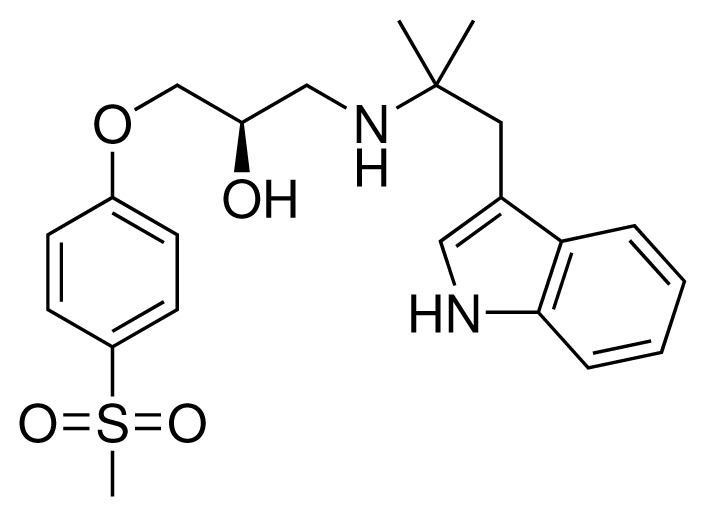	*IC*_50_ = 0.35 μM Δ*G_exp_* = −41.89 kJ mol^−1^ M = 417.55 g mol^−1^ q = +1*e*

**Table 2.** Compounds used in testing of the presented CYP 2D6 LIE model for aryloxypropanolamines. Experimental values for *IC*_50_’s (reported by Vaz *et al.* [27]) and derived binding free energies (Δ*G_exp_*) are given, as well as molar masses (M) and net charges of the compounds (q).

Ligand number # (# in Vaz *et al.*)	Structure	Properties
10 (19)	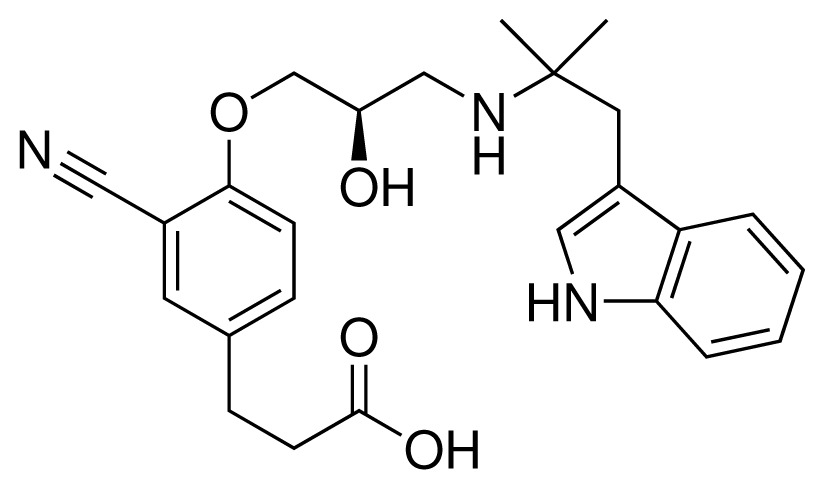	*IC*_50_ = 12 μM Δ*G_exp_* = −32.78 kJ mol^−1^ M = 435.52 g mol^−1^ q = 0*e*
11 (12)	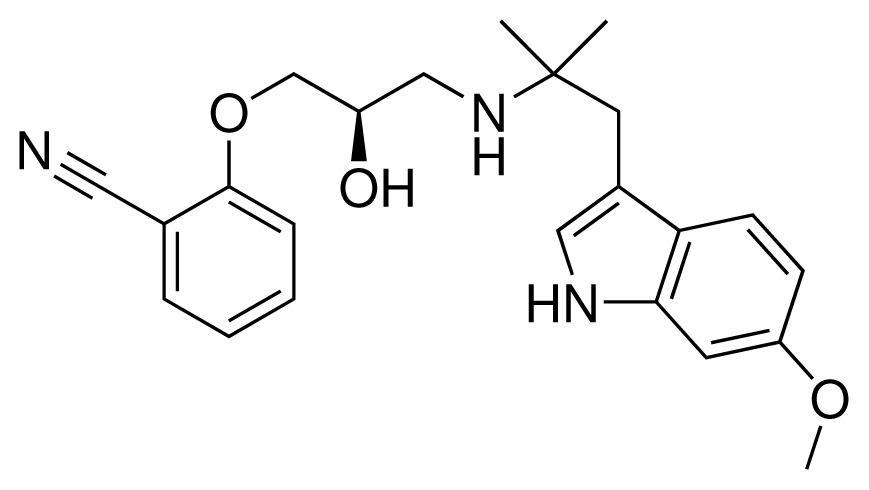	*IC*_50_ = 0.42 μM Δ*G_exp_* = −41.42 kJ mol^−1^ M = 394.50 g mol^−1^ q = +1*e*
12 (16)	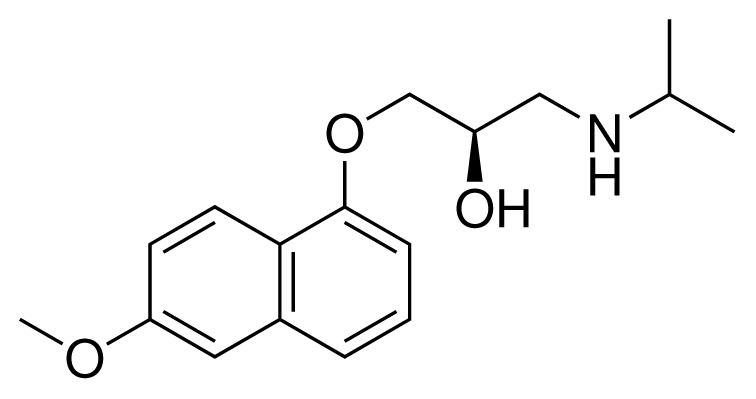	*IC*_50_ = 8.4 μM Δ*G_exp_* = −33.70 kJ mol^−1^ M = 290.38 g mol^−1^ q = +1*e*
13 (18)	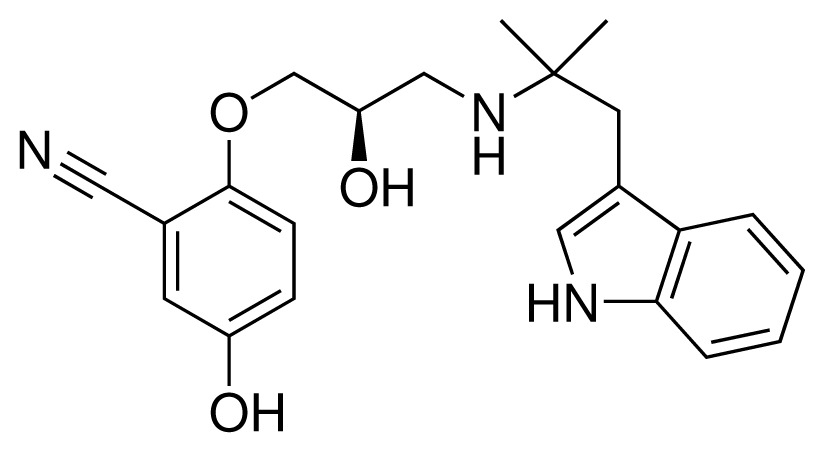	*IC*_50_ = 0.31 μM Δ*G_exp_* = −42.20 kJ mol^−1^ M = 380.47 g mol^−1^ q = +1*e*
14 (14)	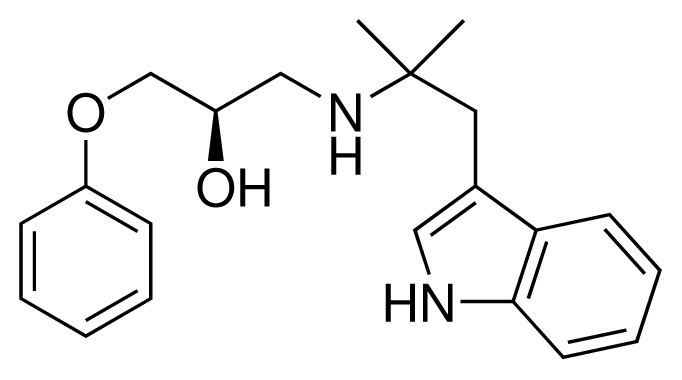	*IC*_50_ = 0.03 μM Δ*G_exp_* = −48.22 kJ mol^−1^ M = 339.46 g mol^−1^ q = +1*e*
15 (4)	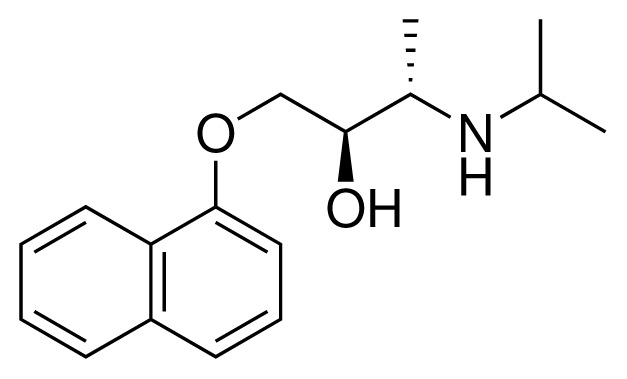	*IC*_50_ = 3.80 μM Δ*G_exp_* = −35.74 kJ mol^−1^ M = 273.38 g mol^−1^ q = +1*e*
16 (9)	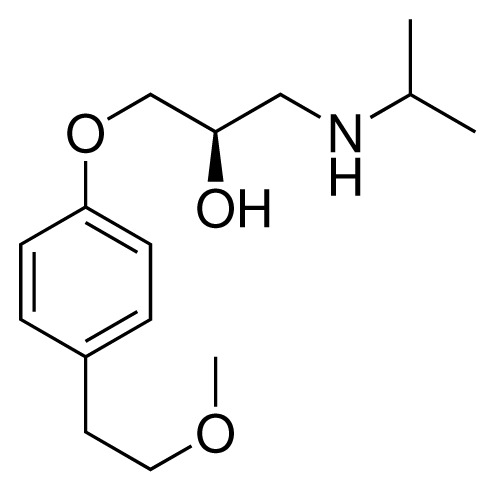	*IC*_50_ = 24 μM Δ*G_exp_* = −30.99 kJ mol^−1^ M = 268.38 g mol^−1^ q = +1*e*
17 (20)	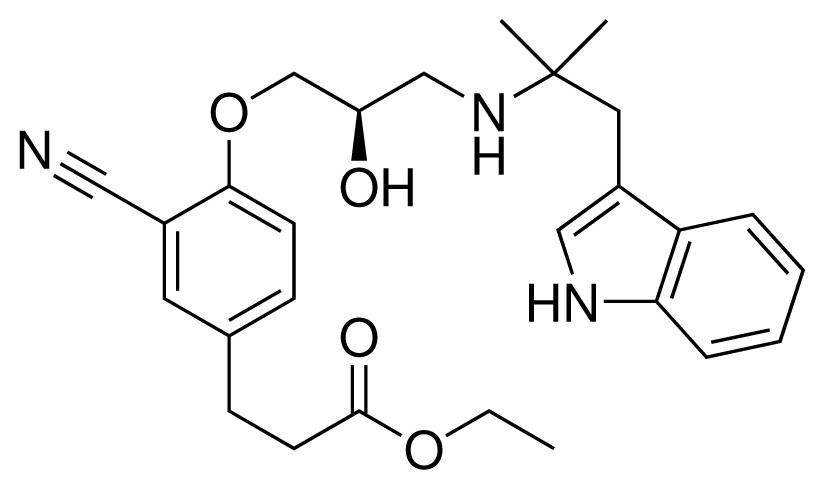	*IC*_50_ = 2.10 μM Δ*G_exp_* = −37.27 kJ mol^−1^ M = 464.59 g mol^−1^ q = +1*e*
