# Supplementary material for: Towards Automated Binding Affinity Prediction Using an Iterative Linear Interaction Energy Approach
Source: Int J Mol Sci. 2014 Jan 9;15(1):798–816. doi: 10.3390/ijms15010798 (PMC3907839; doi:10.3390/ijms15010798)
Supplement: Supplementary file 1 [file ijms-15-00798-s001.pdf]

Supplementary Information

Figure S1. Conformations of the CYP 2D6 active site loop comprising Phe483 in the PPD70 (yellow) and CHZ170 (blue) template structures. The PPD70 and CHZ170 structures were structurally superposed based on fitting of heavy backbone-atom positions. The heme group (green), a bound ligand (compound 3, red) and Phe483 are shown in stick representation.

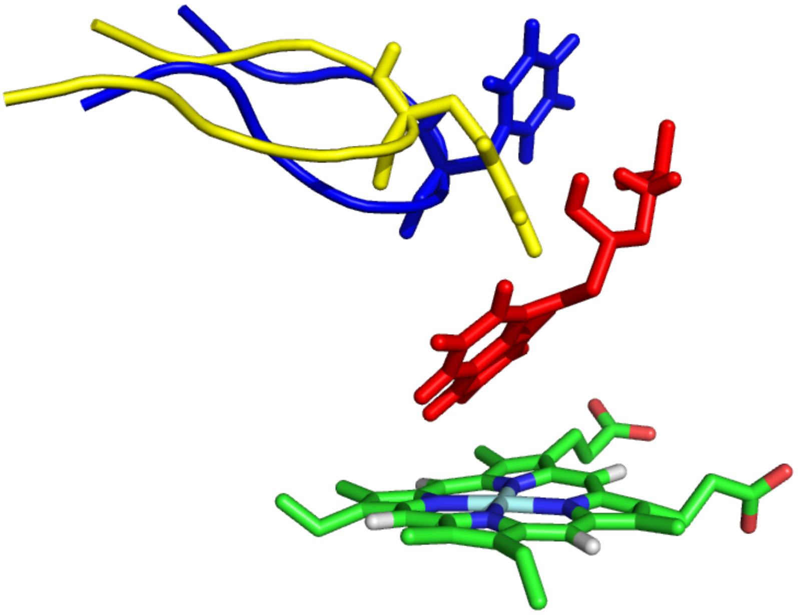

Figure S2. Distribution of values for training (black) and test compounds (red) of the first and second terms on the right-hand side of Equation (3) (designated here as $\alpha \sum_{i=1}^{N} W_i \langle \Delta V_{lig-surr,i}^{vdW} \rangle$ and $\beta \sum_{i=1}^{N} W_i \langle \Delta V_{lig-surr,i}^{el} \rangle$, respectively, in kJ mol^{-1}), as calculated using the CYP 2D6 LIE model that is based on MD simulations starting from all binding conformations in the PPD70 structure. The variances along the model principal axes, as defined in a Mahalanobis or principal component like analysis, are shown as well (σ_0^2 and σ_1^2).

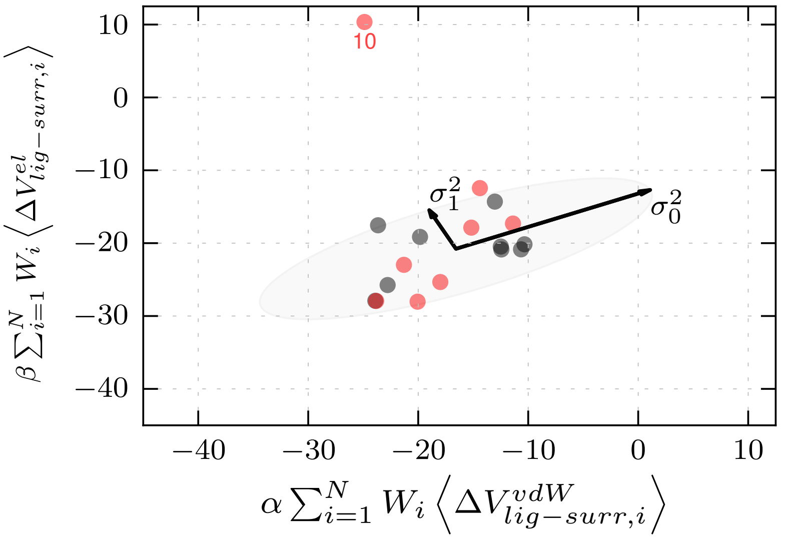

Table S1. Root-mean-square atom-positional deviations (RMSD, in nm) between pairs of ligand-binding poses in CYP 2D6 structures PPD70 (P) and CHZ170 (C), which were used to start MD simulations from. "n.a." indicates pairs of poses that were not used in further analysis. CI refers to the central member of the most populated cluster of docking poses obtained in CHZ170, CII refers to the central member of the second largest cluster of docking poses obtained in CHZ170, *etc*. AVG refers to the average of all RMSD values obtained per ligand. $\Delta\Delta G_{bind}$ refers to individual deviations from experiment of the binding free energy as calculated using the LIE model presented in Figure 3, middle-right panel.

**Compound**	**CI CII**	**CI CIII**	**CII CIII**	**PI PII**	**PI PIII**	**PII PIII**	**CI PI**	**CI PII**	**CI PIII**	**CII PI**	**CII PII**	**CII PIII**	**CIII PI**	**CIII PII**	**CIII PIII**	**AVG**	$\Delta\Delta G_{bind}$ [kJ mol^{-1}]
1	0.38	0.69	0.70	0.27	0.28	0.29	0.26	0.26	0.19	0.30	0.32	0.36	0.72	0.73	0.71	0.43	2.54
2	0.53	0.46	0.62	0.23	0.74	0.73	0.23	0.31	0.66	0.57	0.57	0.36	0.37	0.33	0.79	0.50	2.10
3	0.52	0.66	0.75	0.36	0.84	0.83	0.41	0.30	0.74	0.35	0.48	0.82	0.77	0.73	0.33	0.59	6.09
4	0.63	0.52	0.61	0.69	0.45	0.57	0.39	0.64	0.53	0.48	0.69	0.53	0.53	0.49	0.35	0.54	1.03
5	0.69	0.60	0.63	0.78	0.65	0.55	0.74	0.55	0.55	0.52	0.65	0.47	0.46	0.68	0.70	0.61	5.99
6	0.90	0.78	0.70	0.97	0.48	0.97	0.42	0.88	0.40	0.92	0.61	0.92	0.87	0.39	0.83	0.74	2.01
7	0.86	0.69	0.78	0.92	n.a.	n.a.	0.47	0.89	n.a.	0.90	0.28	n.a.	0.40	0.80	n.a.	0.70	4.76
8	0.82	0.62	0.76	n.a.	n.a.	n.a.	0.74	n.a.	n.a.	0.59	n.a.	n.a.	0.57	n.a.	n.a.	0.68	4.74
9	0.90	0.70	0.63	n.a.	n.a.	n.a.	0.61	n.a.	n.a.	0.79	n.a.	n.a.	0.69	n.a.	n.a.	0.72	0.24
10	0.86	0.60	0.71	n.a.	n.a.	n.a.	0.93	n.a.	n.a.	0.53	n.a.	n.a.	0.86	n.a.	n.a.	0.75	4.81
11	0.84	0.66	0.70	0.90	0.95	0.47	0.78	0.59	0.41	0.50	0.87	0.98	0.74	0.42	0.63	0.70	1.69
12	0.57	0.72	0.64	0.67	0.72	0.35	0.38	0.66	0.71	0.46	0.71	0.68	0.73	0.41	0.27	0.58	1.64
13	0.81	0.80	0.63	0.97	0.40	0.89	0.59	0.76	0.51	0.90	0.43	0.89	0.52	0.76	0.60	0.70	10.48
14	0.90	0.80	0.60	0.90	0.36	0.81	0.42	0.87	0.47	0.88	0.39	0.82	0.85	0.38	0.78	0.68	7.25
15	0.57	0.66	0.54	0.32	0.25	0.37	0.39	0.30	0.47	0.48	0.50	0.33	0.65	0.67	0.59	0.47	3.99
16	0.68	0.54	0.65	0.76	0.31	0.71	0.44	0.68	0.28	0.69	0.40	0.69	0.28	0.71	0.42	0.55	3.74
17	0.82	0.67	0.82	1.00	0.55	1.06	0.56	0.88	0.43	0.86	0.60	0.98	0.53	0.89	0.69	0.76	12.86
